# Coniferous-broadleaf mixed plantations reshape phosphorus-solubilizing bacterial communities and enhance soil phosphorus bioavailability in subtropical forests

**DOI:** 10.48130/forres-0025-0023

**Published:** 2025-10-29

**Authors:** Yuting Zhang, Qiyan Liu, Kai Ding, Qinglin Sun, Meng Lu, Yifan Zhou, Qi Yang, Zaikang Tong, Junhong Zhang

**Affiliations:** State Key Laboratory for Development and Utilization of Forest Food Resources, Zhejiang Key Laboratory of Forest Genetics and Breeding, Zhejiang A&F University, Hangzhou, Zhejiang 311300, China

**Keywords:** Mixed plantations, Soil P fractions, *phoD* and *pqqC* genes, P-solubilizing bacterial communities, P bioavailability

## Abstract

Mixed-species plantations are proven to enhance phosphorus (P) availability in subtropical forest ecosystems. However, the effect of coniferous-broadleaf mixed plantations on soil P cycling dynamics remains poorly understood. Through a long-term field experiment, the study investigated how mixed plantations influence soil P fractions, *phoD* and *pqqC* genes, and associated bacterial communities in bulk and rhizosphere soils. Results showed that compared to monocultures, the introduction of broad-leaved trees significantly increased labile P pools, particularly in the rhizosphere. Amplicon-based community profiling of *phoD*/*pqqC* genes demonstrated distinct compositional shifts in P-solubilizing bacterial communities across forest types and soil compartments. The *pqqC*-harboring bacterial communities were more closely related to the P fractions. More importantly, plant properties were important in explaining bulk soil labile P responses, while in rhizosphere soil, labile P was more strongly associated with soil properties, positively affecting labile P. These findings elucidate the complex interplay between tree diversity, microbial functional traits, and soil P transformations. This study underscores the critical role of mixed plantations in promoting microbial-mediated P mobilization and provides valuable insights for designing sustainable forest management strategies to optimize P utilization in subtropical ecosystems.

## Introduction

Chinese fir (*Cunninghamia lanceolata*), the dominant plantation species in southern China, serves critical roles in timber supply and environmental conservation^[1,2]^. Under the traditional intensive management practices, *C. lanceolata* plantations are often established on the same site for multiple consecutive generations, with a rotation cycle of around 25 years^[3]^. However, long-term planting of *C. lanceolata* leads to decreased stand productivity, soil acidification, and a significant decrease in microbial diversity and soil nutrients^[4]^. The introduction of precious broadleaf species to create coniferous-broadleaf mixed plantations has become an important strategy to improve the productivity of plantations^[5,6]^. A global meta-analysis demonstrates that mixed-species systems based on leaf form complementarity (coniferous and broadleaf) exert significant positive effects on aboveground biomass^[7]^. Previous studies have shown that introducing *Phoebe bournei* into *C. lanceolata* monocultures can effectively facilitate the restoration of soil fertility, especially soil available phosphorus (P)^[8]^. However, the influence of mixed plantations on the composition and dynamics of P-cycling bacterial communities and the transformation processes of soil phosphorus fractions remains poorly understood.

Subtropical *C. lanceolata* plantations exhibit severe P limitation due to Fe/Al oxide-dominated P fixation, with bioavailable P often below 0.5 mg·kg^−^^1^^[9]^. Recent studies highlight the critical role of microbial P cycling in overcoming phosphorus limitation, as soil microorganisms significantly enhance plant P availability by mobilizing both inorganic and organic soil P pools through the secretion of phosphatase enzymes (e.g., via *phoD* genes) and organic acids (mediated by *pqqC*-harboring bacteria)^[10,11]^. The *pqqC* gene, encoding Pyrroloquinoline quinone (PQQ) synthase C required for pyrroloquinoline quinone biosynthesis, represents both a phylogenetic marker for phosphate-solubilizing bacteria and a functional indicator of inorganic P solubilization, mediated through PQQ's role as a cofactor in gluconic acid production^[12]^. The *phoD* gene, which predominates among alkaline phosphatase genes (*phoD, phoA, phoX*) in organic P mineralization pathways, not only encodes the key alkaline phosphatase enzyme in bacteria but also serves as a reliable biomarker for phosphatase activity in terrestrial and aquatic environments^[13,14]^. These microorganisms mediate organic P transformation via community shifts, with *phoD/pqqC* abundance directly correlating with available P^[15]^. For example, Shi et al.^[16]^ found that low nocturnal temperature influenced soil P fractions mainly through changes in the abundance and composition of the *pqqC* gene, with minimal effects on *phoD*.

Soil P exists in multiple chemical forms with varying bioavailability and cycling dynamics. The most effective technique for separating soil P into operationally defined pools is still the Hedley sequential extraction method^[17]^. (1) Labile P (such as resin-P, NaHCO_3_-Pi, and NaHCO_3_-Po), which are readily accessible for plants; (2) moderately labile P (such as NaOH-Pi and NaOH-Po), which act as a reservoir for transitions; and (3) non-labile P (such as HCl-Pi, HCl-Po, and residual-P), which are stable, long-term P reserves^[11,18]^. Importantly, P bioavailability is controlled by the interconversion between these pools, which is facilitated by microbial activity and soil physicochemical characteristics^[19]^. This fractionation technique has been used extensively to measure P pools in afforested soils, offering vital information for determining the P status that plants may access and enhancing afforestation management tactics^[14]^. Notably, relatively labile P may become accessible under particular circumstances (such as pH shifts), whereas labile P pools frequently exhibit strong associations with short-term plant absorption^[20]^. For example, Chen et al.^[21]^ reported that both total and available phosphorus contents initially decreased before increasing with forest age, reaching their lowest levels in middle-aged forests, accompanied by a slow conversion of total P to available P. The application of soil P fractionation techniques allows for precise assessment of P availability, the pool sizes of various P fractions, and the potential for replenishing available P in soil.

While the introduction of *P. bournei* shows promise for alleviating P limitation in degraded *C. lanceolata* plantations, the soil biogeochemical mechanisms—particularly microbial-mediated P cycling processes—remain unresolved. Crucially, it is unknown: (i) how *P. bournei* modifies the stoichiometric balance between organic and inorganic P pools through phosphatase enzyme kinetics and microbial turnover; or (ii) whether mixed cultivation restructures the functional guilds of P-solubilizing bacteria (PSBs), especially core taxa harboring *phoD* and *pqqC* genes. It was hypothesized that: (1) the introduction of *P. bournei* facilitates recalcitrant P mobilization via enhanced enzymatic hydrolysis and microbial turnover; (2) mixed plantations alter soil P cycling by restructuring both the taxonomic diversity of *phoD*- and *pqqC*-harboring bacterial communities and the abundance of these functional genes; and (3) soil labile P pools were regulated by P-solubilizing bacterial communities, plant properties, and soil properties.

## Materials and methods

### Study site and experiment design

The study site was located at Zhujiabu (ZJB) (119°13' E, 29°27' N) field experiment station, Jiande Forestry Farm, in western Zhejiang Province, China. The region has a subtropical monsoon climate. The average annual temperature and precipitation are 16.7 °C and 16,000 mm, respectively. The soil type is Ultisol soils (according to the USDA Soil Taxonomy). The topsoil (0–20 cm) exhibited the following chemical properties: pH 4.76, soil organic matter 32.8 g·kg^−1^, available nitrogen 122.4 mg·kg^−1^, available P 1.2 mg·kg^−1^, and available potassium 42.4 mg·kg^−1^.

The *C. lanceolata* monoculture plantations (planted in 1998 with an initial planting density of 2,500 trees hm^−2^) with generally consistent site conditions, growth conditions, and management history were selected. The experiments were established in the spring of 2015, consisting of eight replicate blocks. Within each block, two 20 cm × 20 m plots with a minimum of 50 m distance between them were randomly assigned to each of two treatments: (1) one control plot was a *C. lanceolata* monoculture, and (2) another plot was mixed plantations (*P. bournei* + *C. lanceolata*): poorly growing trees were removed through thinning (approximately 40% intensity). Subsequently, broadleaf species *P. bournei* (two-year-old seedlings) was introduced for random mixing, in which the ratio of Chinese fir to broadleaf species was about 5:3. During the first two years of reforestation, trees were fertilized with NPK (15-5-12) and nurtured twice a year, after which there was no further intervention. The growth of each tree in each plot was investigated in 2022 (Supplementary Table S1).

### Soil sampling and physicochemical analysis

Soil sampling was conducted in October 2022. From each sample plot, five random soil cores (5 cm diameter × 20 cm depth) were collected, passed through a 2 mm mesh, and combined to form a composite bulk soil sample per plot. The soil surrounding one of the main roots of the target individual was grubbed until a branch with a diameter of less than 2 mm was found, and the above procedure was repeated to collect three complete root branches. The rhizosphere soil was collected by the 'root shaking method'. All soil samples were transported to the laboratory in a refrigerated box immediately after collection. Each composite sample was divided into three portions: one was stored at 4 °C for analysis of P-cycle-related enzyme activities, another at –80 °C for DNA extraction, and the remainder was air-dried for the determination of P fractions and other physicochemical properties.

Total carbon (TC) and nitrogen (TN) contents were analyzed using an elemental analyzer (Vario Max CN, Elementar, Germany). Alkaline phosphatase (ALP) and acid phosphatase (ACP) activities were measured fluorometrically^[22]^. Other physicochemical properties were assessed following the procedures described by Ding et al.^[23]^.

### Soil phosphatase fractions analysis

Soil phosphorus was fractionated using a modified Hedley sequential extraction procedure^[17]^. The detailed extraction protocol is shown in Supplementary Fig. S1. Based on plant bioavailability, the resulting fractions were grouped into three categories: stable P, moderately available P, and labile P. Furthermore, the nine fractions were classified as inorganic (Pi) or organic (Po) phosphorus using the following equations: Pi = Resin-Pi + NaHCO_3_-Pi + HCl-P + HCl-Pi + NaOH-Pi; Po = NaOH-Po + NaHCO_3_-Po + HCl-Po + Residual-P.

### Quantification of *phoD* and *pqqC* genes

Genomic DNA was extracted from approximately 0.50 g of soil employing the PowerSoil® DNA Isolation Kit (MoBio Laboratories, USA). The purity and concentration of the extracted DNA were assessed using a NanoDrop Spectrophotometer (Thermo Fisher Scientific, USA). Absolute quantification of the *phoD* and *pqqC* genes was performed via quantitative real-time PCR (qPCR): ALPS-F730 (5'-CAGTGGGACGACCACGAGGT-3') and ALPS-R1101 (5'-GAGGCCGATCGGCATGTCG-3') for *phoD*^[24]^, Fw (5'-AACCGCTTCTACTACCAG-3') and Rv (5'-GCGAACAGCTCGGTCAG-3') for *pqqC*^[25]^, and F515 (5'-GTGCCAGCMGCCGCGG-3') and R907 (5'-CCGTCAATTCMTTTRAGTTT-3') for 16S rRNA^[26]^. The amplification system was the Light Cycler 480 System (Roche, Basel, Switzerland), with three technical replicates per sample. The cloned plasmid was diluted ten times, and the standard curve was plotted and the number of copies of the gene expressed per gram of dry soil was obtained from the standard curve.

### High-throughput sequencing and bioinformatics analysis

The bacterial *phoD* and *pqqC* genes were amplified and sequenced using the primer pairs ALPSF730/ALPSR1101 and Fw/Rv, respectively. PCR amplicons were purified with the E.Z.N.A.® Gel Extraction Kit (Omega, USA) and sequenced on an Illumina Nova6000 platform (Guangdong Magigene Biotechnology Co., Ltd., China).

Raw sequences were processed in QIIME2^[27]^ to remove reads containing ambiguous bases and those with quality scores below 20. Chimeras were filtered using the DADA2 pipeline^[28]^ to generate high-quality amplicon sequence variants (ASVs). Taxonomic assignment of representative ASVs was performed against the NCBI nucleotide database via BLAST (www.ncbi.nlm.nih.gov) and usearch-sintax at a confidence threshold of 0.8. A total of 2,877,210 (range 50,310–86,700) high-quality filtered *phoD*-harboring bacterial sequences and 3,886,476 (range 75,193–110,892) *pqqC*-harboring bacterial sequences were obtained across all soil samples. The ASV table was rarefied to the minimum sequence depth per sample for subsequent comparative analyses.

### Statistical analysis

To evaluate the effects of forest type, soil compartment, and their interaction on soil P fractions, physicochemical properties, enzyme activities, microbial diversity, and functional gene abundance, linear mixed models (LMMs) were fitted using the 'lmer' function in the 'lme4' and 'lmerTest' packages^[29]^, with field block included as a random effect. One-way ANOVA followed by Tukey's HSD test (*p* < 0.05) was conducted in SPSS (v22.0, IBM, USA). The natural log-transformed response ratio (RR) was calculated to quantify the effect size of tree species mixtures on each variable. Beta-diversity patterns across bulk and rhizosphere soils under different forest types were visualized using non-metric multidimensional scaling (NMDS) based on Bray–Curtis distances using the 'vegan' package. Permutational multivariate analysis of variance (Adonis) was applied to test for significant differences in P-solubilizing bacterial community structure. Associations between soil P fractions and bacterial community characteristics were assessed by Mantel tests using the 'linkET' package. To further elucidate relationships between P fractions and bacterial community composition, distance-based redundancy analysis (dbRDA) was performed based on Bray–Curtis dissimilarities using the 'vegan' package. Hierarchical partitioning analysis was subsequently employed to quantify the independent contributions of each P fraction using the 'rdacca.hp' package^[30]^.

To assess the effects of tree species mixtures on labile P, a generalized linear mixed model (GLMM) was fitted using the 'lme4' package. Predictor variables were grouped into three categories: plant properties, soil properties, and microbial properties. Plot identity was incorporated as a random effect to control for spatial autocorrelation, while all variables were included as fixed effects. To address multicollinearity, variance inflation factors (VIF) were calculated via the 'vif' function in the 'ca' package, iteratively excluding variables with VIF > 10 until all retained predictors met the threshold. The 'dredge' function in the 'MuMIn' package was then used to generate all possible candidate models ranked by corrected Akaike's Information Criterion (AICc). Model averaging was applied to top-ranked models (ΔAICc < 2) to identify robust fixed-effect predictors. Finally, the relative importance of each predictor was evaluated using the 'glmm.hp' package^[31]^. Then, hypothesis-driven structural equation modelling was developed using the 'piecewiseSEM' package (SEM), and a priori conceptual framework based on ecological theory postulates that plant properties, soil properties, and microbial properties will regulate phosphorus bioavailability (Supplementary Fig. S2). Model adequacy was verified through multi-criteria evaluation, including Fisher's C statistic (*p* > 0.05) and comparative Akaike Information Criterion (AIC) values.

## Results

### Soil phosphorus fractions and physicochemical characteristics

The findings revealed significant variations in soil P fractions across forest types and soil compartments (bulk vs rhizosphere) (Table 1). Specifically, mixed plantations significantly increased the contents of all P fractions in bulk soil (*p* < 0.05), except for HCl-Po and Residual-P. In rhizosphere soil, mixed plantations also markedly enhanced all P fractions, with the most pronounced effects observed in RMP.

**Table 1:** Soil P fractions, physicochemical properties, and enzyme activities in bulk soil and rhizosphere soil.

Parameters	BM	BP	RMC	RMP	RPC	*p*-value of linear mixed models			
						Forest type	Soil compartments	Type × compartments	Block
Labile P									
Resin-P (mg·kg^−1^)	6.03 ± 0.81 d	6.20 ± 0.69 d	12.64 ± 0.92 c	15.54 ± 1.02 a	14.27 ± 0.75 b	0.500	**< 0.001**	**< 0.001**	0.994
NaHCO_3_−Pi (mg·kg^−1^)	12.98 ± 1.91 b	10.64 ± 0.70 b	39.36 ± 2.85 a	39.63 ± 2.06 a	37.11 ± 1.71 a	0.628	**< 0.001**	**< 0.001**	0.995
NaHCO_3_−Po (mg·kg^−1^)	51.40 ± 5.61 a	26.25 ± 3.09 c	42.64 ± 3.53 b	49.03 ± 2.45 a	38.11 ± 3.55 b	**< 0.001**	0.655	**< 0.001**	0.934
Moderate labile P									
NaOH-Pi (mg·kg^−1^)	22.21 ± 1.84 d	19.03 ± 1.70 d	40.93 ± 3.08 b	48.36 ± 2.27 a	35.72 ± 2.47 c	0.199	**< 0.001**	**< 0.001**	0.976
NaOH-Po (mg·kg^−1^)	150.22 ± 6.01 a	92.52 ± 2.92 d	104.92 ± 4.35 c	129.25 ± 6.75 b	102.22 ± 5.47 c	**< 0.001**	**0.021**	**< 0.001**	0.987
HCl-P (mg·kg^−1^)	7.85 ± 0.93 c	6.91 ± 0.82 c	17.42 ± 1.58 b	22.37 ± 1.13 a	18.42 ± 1.71 b	0.989	**< 0.001**	**< 0.001**	0.991
Stable P									
HCl-Pi (mg·kg^−1^)	172.21 ± 10.04 c	121.45 ± 4.06 d	162.67 ± 3.83 c	199.68 ± 8.69 b	225.26 ± 5.30 a	0.655	**< 0.001**	**< 0.001**	0.999
HCl-Po (mg·kg^−1^)	110.65 ± 14.92 b	318.32 ± 19.03 a	67.48 ± 7.93 c	81.98 ± 4.43 c	81.89 ± 4.08 c	**< 0.001**	**< 0.001**	**< 0.001**	0.996
Residual-P (mg·kg^−1^)	87.53 ± 3.03 c	99.74 ± 5.15 a	99.40 ± 6.61 a	97.11 ± 4.02 ab	90.85 ± 4.57 bc	0.464	0.550	**< 0.001**	0.782
Chemical properties									
pH	4.63 ± 0.06 a	4.54 ± 0.06 b	4.39 ± 0.13 c	4.62 ± 0.04 a	4.42 ± 0.08 bc	0.337	**< 0.001**	**< 0.001**	0.892
SWC (%)	17.52 ± 0.76 a	17.56 ± 1.88 a	15.06 ± 1.06 b	15.22 ± 0.95 b	14.97 ± 1.19 b	0.968	**< 0.001**	**< 0.001**	0.707
AN (mg·kg^−1^)	127.75 ± 17.55 b	105.00 ± 13.36 c	149.80 ± 15.81 a	158.20 ± 14.90 a	100.10 ± 12.07 c	**< 0.001**	0.312	**< 0.001**	0.699
SOM (g·kg^−1^)	31.58 ± 3.58 bc	25.88 ± 3.81 cd	41.86 ± 5.18 a	37.46 ± 4.71 ab	23.22 ± 4.22 d	**< 0.001**	0.181	**< 0.001**	0.859
TC (g·kg^−1^)	17.13 ± 2.05 b	12.43 ± 2.19 c	23.02 ± 3.42 a	21.59 ± 2.52 a	14.47 ± 3.56 bc	**< 0.001**	**0.015**	**< 0.001**	0.930
TN (g·kg^−1^)	1.44 ± 0.18 ab	1.21 ± 0.16 b	1.70 ± 0.28 a	1.68 ± 0.17 a	1.18 ± 0.22 b	**< 0.001**	0.250	**< 0.001**	0.836
TP (g·kg^−1^)	0.58 ± 0.04 c	0.66 ± 0.06 bc	0.63 ± 0.10 bc	0.84 ± 0.06 a	0.68 ± 0.03 b	**0.003**	0.097	**0.005**	0.096
C/N	11.92 ± 0.57 b	10.24 ± 1.03 c	13.55 ± 0.58 a	12.84 ± 0.37 ab	12.15 ± 0.77 b	**0.001**	**< 0.001**	**< 0.001**	0.865
C/P	29.30 ± 0.57 b	19.07 ± 1.03 d	36.62 ± 0.58 a	25.84 ± 0.37 bc	21.10 ± 0.77 cd	**< 0.001**	0.088	**< 0.001**	0.743
Biological characters									
ALP (nmol·(g·h)^–1^)	378.24 ± 39.48 c	386.23 ± 44.22 c	630.63 ± 41.25 a	658.85 ± 65.02 a	543.96 ± 62.02 b	0.334	**< 0.001**	**< 0.001**	0.814
ACP (nmol·(g·h)^–1^)	1713.06 ± 168.92 b	1011.64 ± 219.72 c	2456.53 ± 99.30 a	2543.47 ± 23.69 a	2384.93 ± 41.21 a	0.062	**< 0.001**	**< 0.001**	0.975
Data are means ± SD (n = 8). Values followed by different letters within a column indicate significant differences based on one-way ANOVA (*p* < 0.05). SWC, soil water content; AN, available nitrogen; SOM, soil organic matter; TC, total carbon; TN, total nitrogen; TP, total phosphorus; C/N, total carbon to total nitrogen ratio; C/P, total carbon to total phosphorus ratio; ALP, alkaline phosphatase; ACP, acid phosphatase. BM, bulk soil in mixed plantations; BP, bulk soil in monocultures; RMC, rhizosphere soil of *Cunninghamia lanceolata* in mixed plantations; RMP, rhizosphere soil of *Phoebe bournei* in mixed plantations; RPC, rhizosphere soil of *Cunninghamia lanceolata* in monocultures. Bold values denote significant differences (*p* < 0.05).									

**Figure 1:**
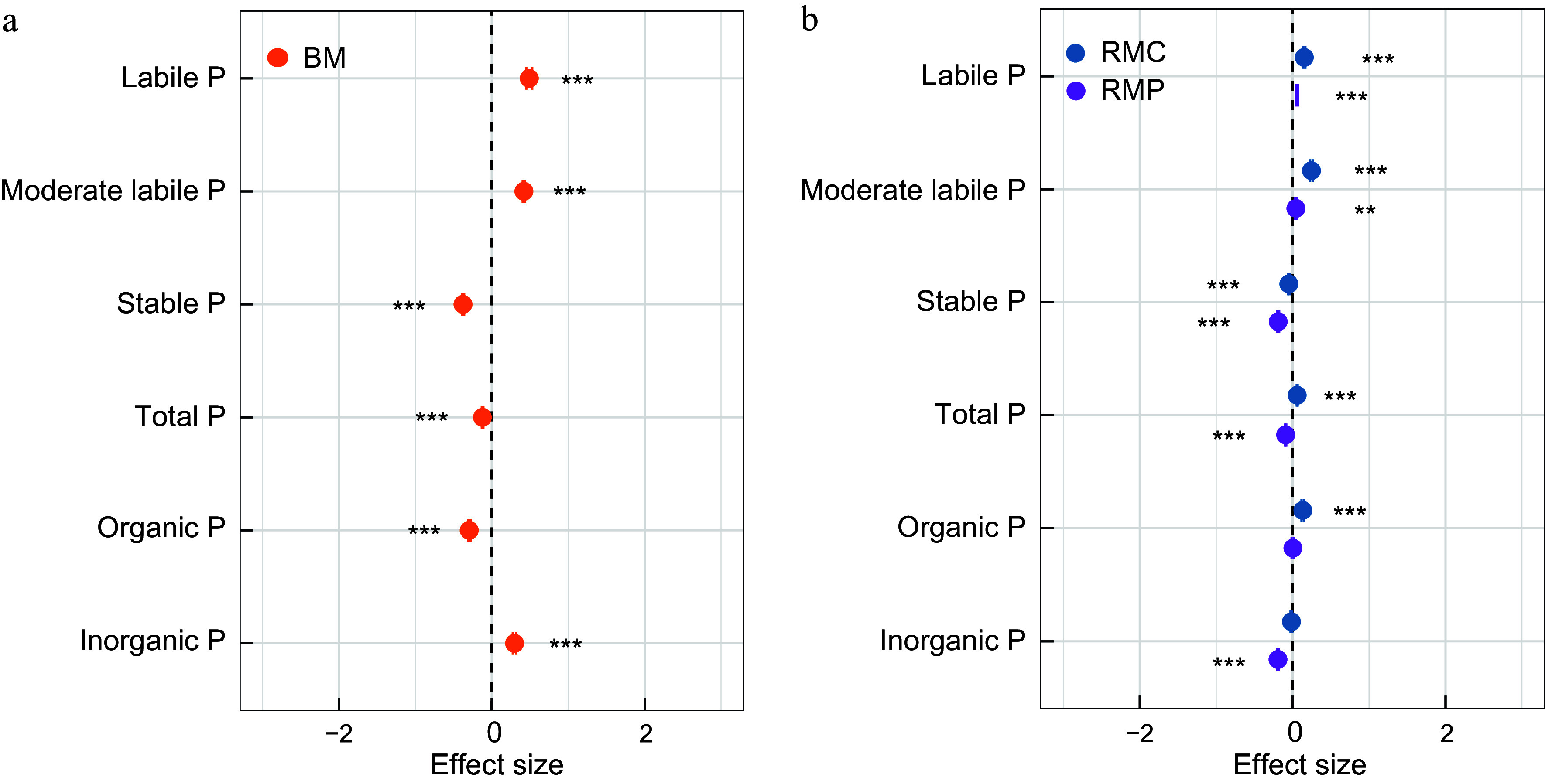
Effects of tree species mixtures on the P fractions in (a) bulk, and (b) rhizosphere soil. Data are presented as means ± 95% confidence intervals. Asterisks indicate significant mixture effects: ** *p* < 0.01; *** *p* < 0.001. BM, bulk soil in mixed plantations; RMC, rhizosphere soil of *Cunninghamia lanceolata* in mixed plantations; RMP, rhizosphere soil of *Phoebe bournei* in mixed plantations.

Compared to monocultures, mixed plantations had higher labile P concentrations (Fig. 1; Supplementary Table S2), including Resin-P, NaHCO_3_-Pi, and NaHCO_3_-Po, particularly in rhizosphere soils. The highest labile P levels were observed in RMP. Moderate labile P followed a similar trend, with mixed plantations showing elevated values relative to monocultures. In contrast, stable P pools, organic P, and total P were reduced in mixed plantations. Notably, bulk soil in monocultures had the highest proportion of Po, accounting for 76.56% of total P, whereas mixed plantations contained only 64.37%. The relative proportions of Po and Pi in rhizosphere soils showed a similar pattern (Supplementary Fig. S3). Furthermore, mixed plantations significantly improved soil physicochemical properties such as pH, SOC, TC, and TN as well as ACP activity, with a more pronounced effect in the rhizosphere soils (Table 1). However, mixed plantations had a minor effect on SWC and ALP in bulk soils.

### Abundance and diversity of *phoD* and *pqqC* genes

**Figure 2:**
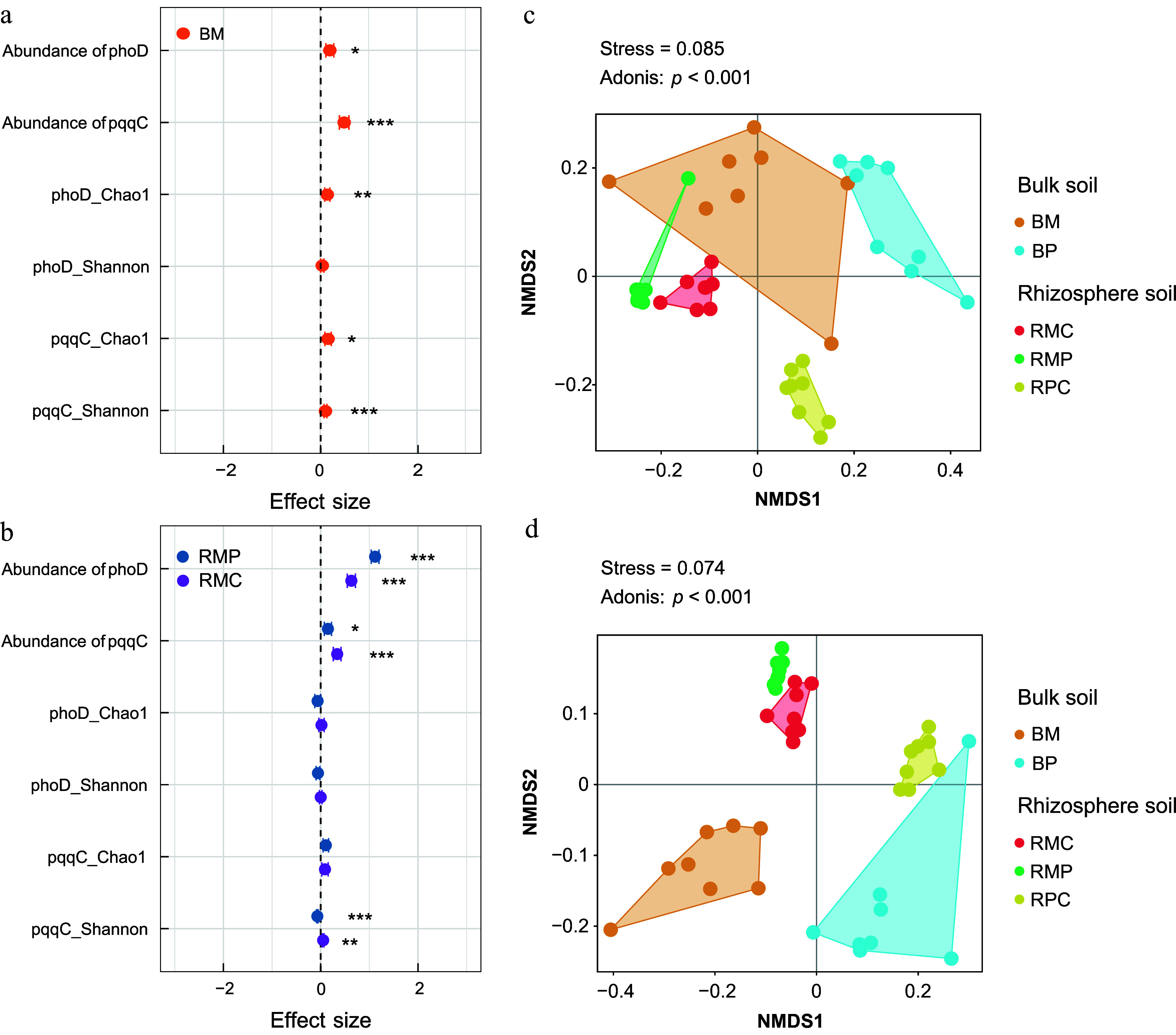
Effects of tree species mixtures on gene abundance and diversity of *phoD*- and *pqqC*-harboring bacterial communities in (a) bulk, and (b) rhizosphere soils. Data represent means ± 95% confidence intervals. Significant differences are marked with asterisks: * *p* < 0.05; ** *p* < 0.01; *** *p* < 0.001. NMDS ordination based on Bray–Curtis dissimilarity illustrating compositional shifts in (c) *phoD*- and (d) *pqqC*-harboring communities across forest types and soil compartments. BM, bulk soil in mixed plantations; BP, bulk soil in monocultures; RMC, rhizosphere soil of *Cunninghamia lanceolata* in mixed plantations; RMP, rhizosphere soil of *Phoebe bournei* in mixed plantations; RPC, rhizosphere soil of *Cunninghamia lanceolata* in monocultures.

Compared to monocultures, mixed plantations significantly enhanced the abundance of both *pqqC* and *phoD* genes in bulk and rhizosphere soils (Fig. 2a, b; Supplementary Table S3). Similarly, mixed plantations markedly increased the alpha diversity (Shannon and Chao1 indices) of *pqqC*-harboring bacterial communities in bulk soil (*p* < 0.05), while only the Chao1 index of *phoD*-harboring communities was significantly elevated in bulk soil (Fig. 2a, b). In rhizosphere soil, forest type exerted a significant influence on the Shannon diversity of *pqqC*-harboring communities. Non-metric multidimensional scaling (NMDS) further indicated significant differences in the beta diversity of both *phoD*- and *pqqC*-harboring communities across forest types and soil compartments (*p* < 0.001; Fig. 2c, d).

### P-solubilizing bacterial community composition

**Figure 3:**
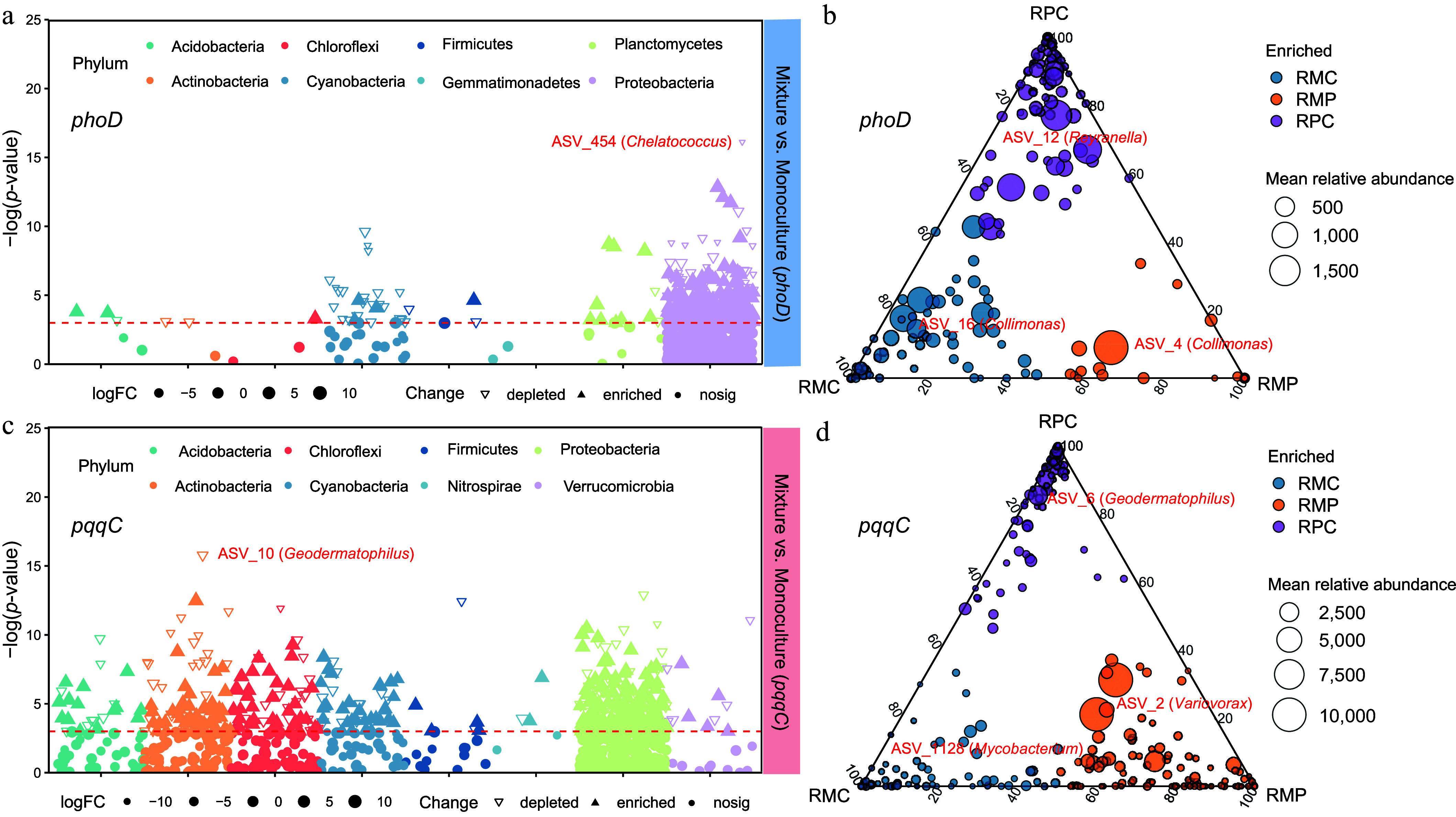
Manhattan and ternary plots of enriched ASVs in *phoD*- and *pqqC*-harboring bacterial communities within (a), (c) bulk, and (b), (d) rhizosphere soils across forest types. Point size corresponds to mean relative abundance. Colors indicate significant enrichment (Kruskal–Wallis test, *p* < 0.05). BM, bulk soil in mixed plantations; BP, bulk soil in monocultures; RMC, rhizosphere soil of *Cunninghamia lanceolata* in mixed plantations; RMP, rhizosphere soil of *Phoebe bournei* in mixed plantations; RPC, rhizosphere soil of *Cunninghamia lanceolata* in monocultures.

Manhattan plots showed that the relative abundance of several *phoD*- and *pqqC*-harboring bacterial ASVs in bulk soil differed between monocultures and mixed plantations (*p* < 0.05), with ASV_454 and ASV_10 especially showing the greatest differences (Fig. 3a, c). Ternary plots revealed that *phoD*-harboring bacterial were ASV_16, ASV_4, and ASV_12 was enriched in RMC, RMP, and RPC rhizosphere soils, respectively (Fig. 3b). The *pqqC*-harboring bacterial ASV_1128, ASV_2, and ASV_6 were enriched in RMC, RMP, and RPC rhizosphere soils, respectively (Fig. 3d).

The bacterial communities harboring *phoD* and *pqqC* genes were primarily dominated by Proteobacteria, Actinobacteria, Verrucomicrobia, and Chloroflexi, accounting for more than 80% (Supplementary Fig. S4a, c). Furthermore, *Reyranella*, *Collimonas*, *Bradyrhizobium*, *Chelatococcus*, *Pseudolabrys*, *Beijerinckia*, *Hydrocarboniphaga*, and *Ramlibacter* were the predominant genera of the *phoD*-harboring communities (Supplementary Fig. S4b). *Chthoniobacter*, *Paraburkholderia*, *Variovorax*, *Geodermatophilus*, *Mastigocoleus*, *Amycolatopsis*, *Steroidobacter*, and *Reticulibacter* were the predominant genera of the *pqqC*-harboring communities (Supplementary Fig. S4d).

### Relationships between the characteristics of P-solubilizing bacterial communities and soil P fractions

**Figure 4:**
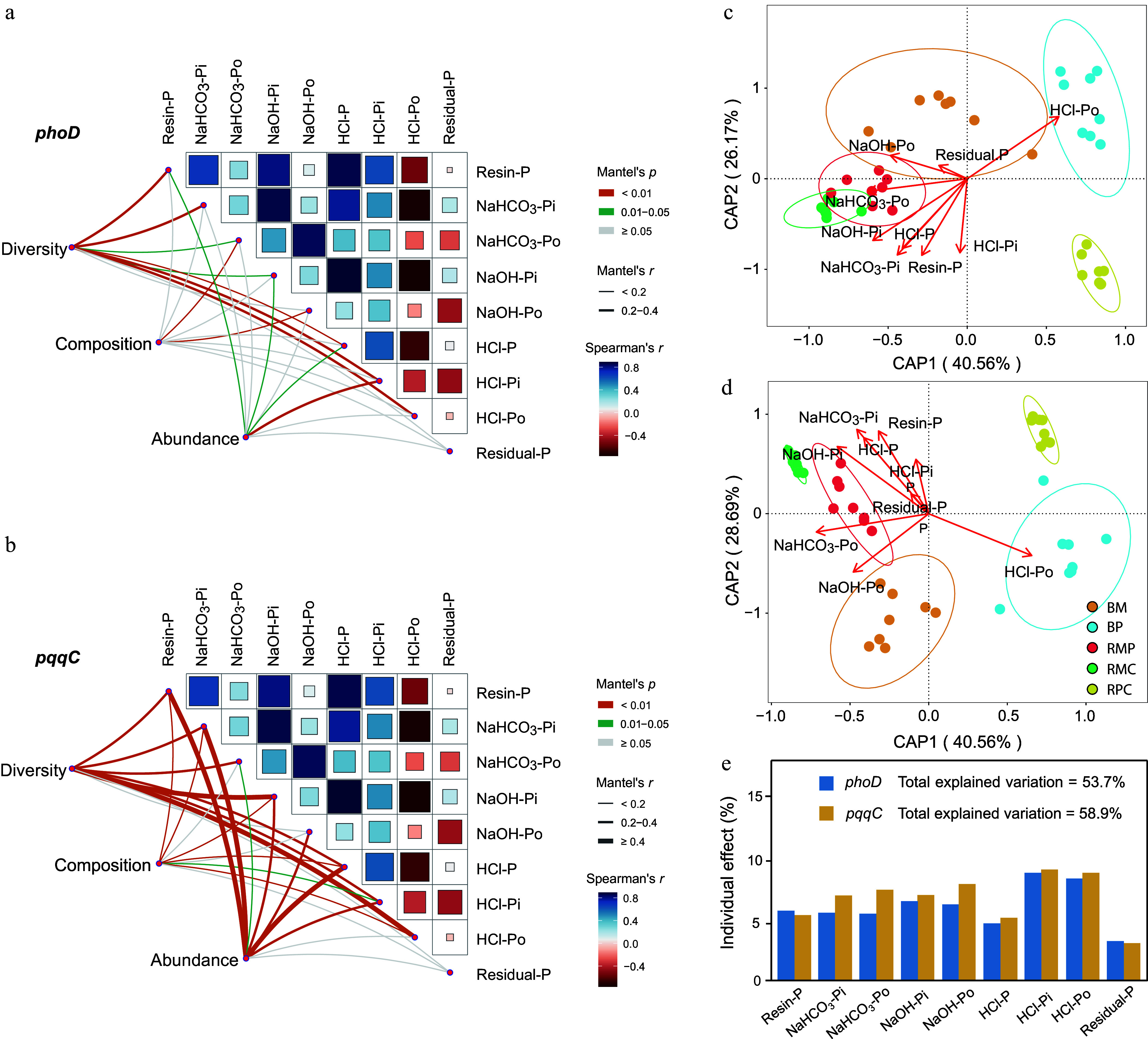
Relationships between (a), (c) *phoD-, *and (b), (d) *pqqC*-harboring bacterial communities and soil P fractions, evaluated using Mantel tests and distance-based redundancy analysis (dbRDA) with Bray–Curtis dissimilarity. Percentages in parentheses indicate variance explained per axis. (e) Bar plot shows individual contribution of each P fraction.

Soil P fractions were regulated by the *phoD-* and *pqqC-*harboring bacterial communities (Fig. 4a, b). The diversity and abundance of the two genes were more closely related to the P fractions compared to the composition, especially for *pqqC*. The dbRDA results indicated that soil P fractions accounted for 66.73% and 70.06% of the variance in community composition of *phoD*- and *pqqC*-harboring microorganisms, respectively (Fig. 4c, d). Among these fractions, HCl-Pi made the greatest contribution, explaining 8.24% and 8.94% of the variance, respectively (Fig. 4e).

**Figure 5:**
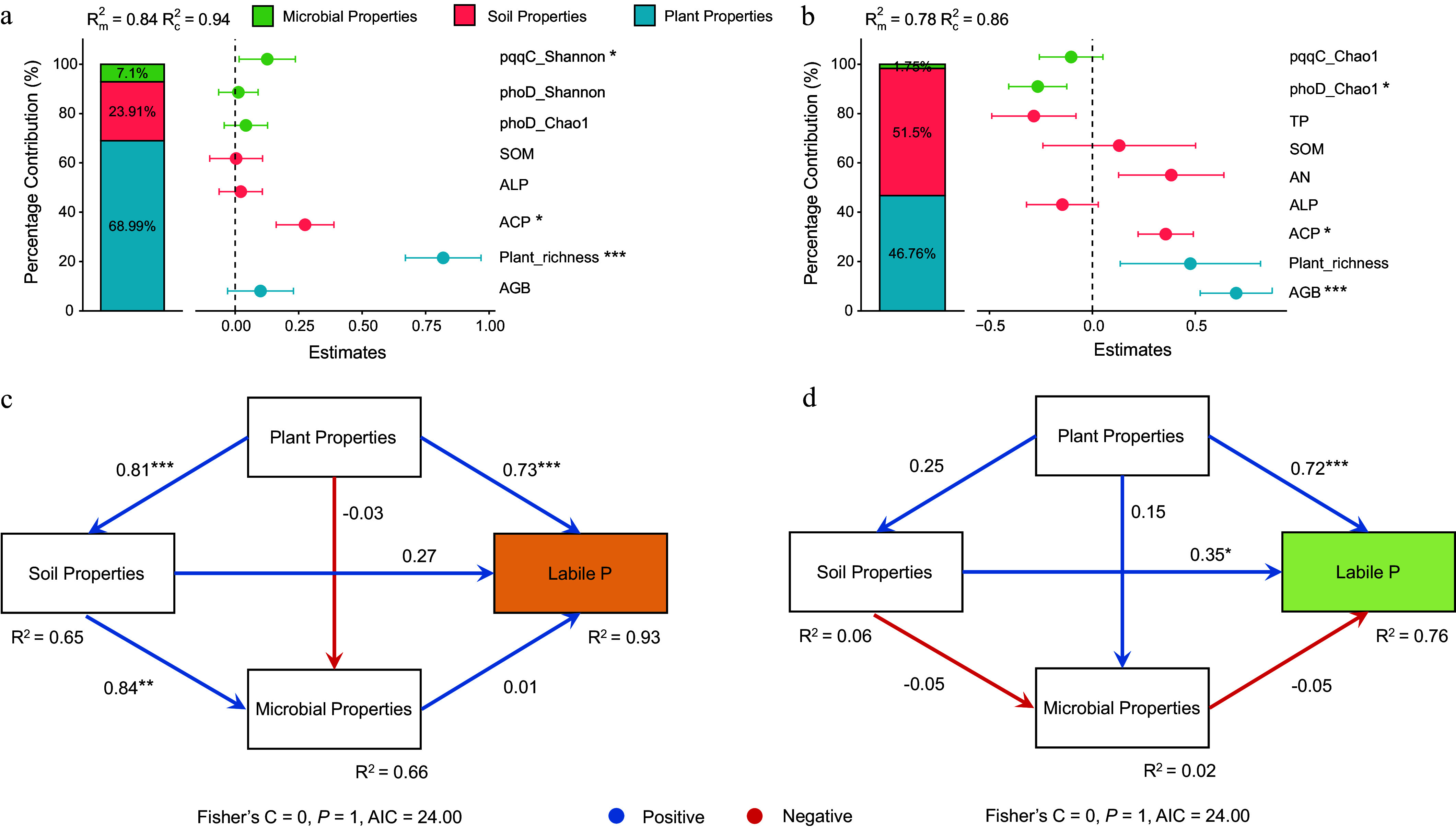
Optimal models depicting effects of plant properties, soil properties, and microbial traits on labile P in (a) bulk, and (b) rhizosphere soils. Bars represent mean parameter estimates with 95% confidence intervals. Piecewise structural equation models (SEM) illustrate direct and indirect effects on labile P in (c) bulk, and (d) rhizosphere soils. Values below boxes denote explained variance (R^2^); path coefficients are shown alongside arrows. Significance levels: * *p* < 0.05; ** *p* < 0.01; *** *p* < 0.001. AGB: aboveground biomass; AN: available nitrogen; SOM: soil organic matter; TP: total phosphorus; ALP: alkaline phosphatase; ACP: acid phosphatase.

Plant, soil, and microbial properties explained 68.99%, 23.91%, and 7.10% of the variation in labile P in bulk soil (Fig. 5a), and 46.76%, 51.50%, and 1.75% in rhizosphere soil (Fig. 5b), respectively. In bulk soil, plant richness, ACP, and the Shannon diversity of *pqqC*-harboring bacterial communities were significantly positively correlated with labile P (*p* < 0.05). In the rhizosphere soil, AGB and ACP showed positive correlations with labile P, whereas the Chao1 richness of *phoD*-harboring bacterial communities was negatively correlated. SEM further revealed that plant properties exerted significant direct positive effects on labile P in both bulk and rhizosphere soils, as well as on soil properties in bulk soil (*p* < 0.05; Fig. 5c, d). Soil properties positively influenced both microbial properties and labile P in the rhizosphere soil (*p* < 0.05).

## Discussion

### The effect of mixed plantations on soil bioavailable P

P is an important nutrient for plant growth and metabolism, participates in biogeochemical cycles, and is a major factor in soil productivity^[10,11,32]^. A large proportion of total P exists in organic forms, serving as the primary P source and accounting for over 80% of all P in *C. lanceolata* plantation ecosystems^[33]^. Consistent with this study (Supplementary Fig. S3), bulk soil in monocultures had a similar proportion of organic P. Phosphatase-mediated hydrolysis of organic P into inorganic forms is essential for plant P uptake^[34]^ and reflects microbial nutrient demand and metabolic activity^[35]^. Both ACP and ALP activities were significantly higher in the rhizosphere than in the bulk soil (Table 1), suggesting that enhanced phosphatase activity in the rhizosphere may facilitate more rapid mineralization of organic P in this microenvironment. This conclusion aligns with the soil inorganic P/organic P ratio, demonstrating that higher soil phosphatase activity corresponds to increased inorganic P content (Supplementary Fig. S3). This may be due to the fact that broadleaf species have more complex root morphology and secretions, resulting in greater enzyme activity. The higher nutrient content of mixed plantation soils provides sufficient C and N sources for microorganisms^[23]^, which stimulates microbial activity and increases soil enzyme activity, thus may contribute to promoting soil Po content.

Characterization of soil P fractions is fundamental to deciphering P cycling processes, given their role as reservoirs of potentially bioavailable P^[36]^. Compared with monocultures, mixed plantations significantly enhanced labile P while reducing residual P, resulting in a higher proportion of labile and moderately labile phosphorus fractions (Table 1). Soil total P increased significantly during forest succession, driven by enhanced tree diversity, litter input, and soil organic carbon accumulation in mixed plantations^[37]^. As succession progressed, the forest P cycle transitioned from acquisition to recycling, ultimately promoting greater plant P uptake^[38]^. Notably, labile P content was higher in rhizosphere soils of different stands than in bulk soils (Fig. 1). Mixed plantations foster diverse root architectures and exudation profiles, which act as substrates for P-solubilizing microorganisms, stimulating their activity and promoting the mineralization of Po and dissolution of Pi via acidification or chelation^[39]^.

Mixed plantations improve soil structure and organic matter (SOM) accumulation, fostering microenvironments that favor P retention and microbial activity. For instance, diverse litter inputs increase SOM heterogeneity, which may stabilize labile P via organo-mineral associations while providing C sources for P-solubilizing microbes^[40−42]^. Higher levels of SOC, TC, and TN in mixed plantations were associated with soil P availability (Table 1). In line with this, Hou et al.^[43]^ showed that SOC content was positively correlated with P content, especially Po content. If the soil contains a high level of SOC, the soluble C and other intermediates produced by its decomposition can trigger the reaction and adsorption of P in the soil, and ultimately regulate the dynamics of the P fractions^[44]^.

### The effect of mixed plantations on the abundance and P-solubilizing microbial communities

The *phoD* gene, encoding alkaline phosphatase, facilitates Po mineralization, while *pqqC* regulates the synthesis of pyrroloquinoline quinone (PQQ), a cofactor for phosphate-solubilizing enzymes^[45,46]^. The observed elevation in *phoD* and *pqqC* gene abundances—particularly the pronounced enrichment of *pqqC—*in mixed plantations highlights a functional prioritization of microbial P mobilization strategies under tree diversity (Fig. 2), as revealed by the results of the Mantel test (Fig. 4b). Monocultures exhibited lower microbial biomass and stronger phosphorus limitation, consequently reducing the abundance of *phoD* and *pqqC* genes. In contrast, the introduction of broadleaf species in mixed plantations alleviated P limitation, supporting higher gene abundances^[3]^. On the other hand, subtropical soils are often acidic, favoring Pi solubilization via proton extrusion–a process enhanced by PQQ-dependent dehydrogenases (*pqqC*)^[47]^. Lower pH in monoculture plantations may also inhibit *phoD*-encoded alkaline phosphatase activity, probably explaining the relatively smaller increase in *phoD* abundance compared to *pqqC* (Fig. 2; Table 1). Moreover, mixed-species litter inputs increase organic matter heterogeneity, providing varied C substrates (e.g., lignin, cellulose) that select for decomposer communities with distinct P-acquisition strategies^[40,48]^. High lignin content in some litter may favor *pqqC*-harboring Actinobacteria, which excel at solubilizing Pi while decomposing complex C^[49]^, whereas *phoD*-harboring Proteobacteria may dominate in litter with labile C.

### P-solubilizing bacterial communities drove the improvement in soil labile P

Soil P-solubilizing microorganisms mediate the turnover of organic and microbial biomass P, as well as the mobilization of fixed inorganic phosphorus^[16,50]^. The observed regulation of soil P fractions by *phoD*- and *pqqC*-harboring bacterial communities, particularly through their diversity and abundance rather than compositional shifts, especially for *pqqC* (Fig. 4). On the one hand, diverse *phoD* or *pqqC* communities may harbor taxonomically distinct but functionally equivalent members capable of performing similar P-mobilizing roles^[51]^. For example, various *phoD*-carrying taxa (e.g., Actinobacteria, Proteobacteria) can produce alkaline phosphatases, ensuring stable organic P mineralization even if community composition shifts^[52]^. On the other hand, the *pqqC*-encoded PQQ activates glucose dehydrogenase (GDH) in PSBs^[12]^, leading to the production of gluconic acid. This compound acidifies the rhizosphere and chelates Fe^3+^/Al^3+^, thereby solubilizing mineral-bound phosphorus^[53]^. Previous studies showed that potential keystone taxa such as *Geodermatophilus* exhibited a significant positive correlation with maize biomass^[54]^. Therefore, increased abundance of ASV_10 (*Geodermatophilus*) in *pqqC*-harboring bacterial communities may contribute to more efficient phosphorus acquisition in monoculture plantations on nutrient-poor soils (Fig. 3).

The SEM further showed that plant properties were important in explaining bulk soil labile P responses, while in rhizosphere soil, labile P was more strongly associated with soil properties (Fig. 5). This divergence may be attributed to the distinct ecological niches and influencing mechanisms in the two soil compartments. In bulk soil, plant-derived inputs (such as root exudates and litter) serve as the primary source of labile organic P^[55]^, while root-induced alterations to soil structure and moisture further enhance P mobilization. In contrast, the rhizosphere represents a hotspot for plant–microbe–soil interactions, where soil properties (e.g., pH and enzyme activities) become dominant in regulating P solubility and stability^[14]^. The stronger influence of soil properties in the rhizosphere likely reflects intensified biogeochemical processes driven by root-induced changes in microbial community composition and function^[10,16]^.

Collectively, while the results clearly demonstrate the benefits of mixing *C. lanceolata* with *P. bournei*, an important question is whether these patterns are generalizable across other species combinations and ecosystems. The key drivers-enhanced rhizosphere effects from diverse root architectures, increased input of organic matter, and the subsequent stimulation of P-solubilizing microbial communities-are fundamental ecological processes not unique to this species pair. For instance, other coniferous-broadleaf mixtures (e.g., *C. lanceolata* with *Quercus gilva*. or *P. zhennan*) in subtropical regions also exhibit similar improvements in soil fertility and microbial activity, suggesting a parallel potential for microbial-mediated P mobilization. Future research should test these mechanisms across a gradient of species pairs and soil types to establish the broader ecological applicability of our model.

## Conclusions

The findings of this study demonstrated that mixed plantations in subtropical forests enhanced soil P availability by reshaping microbial functional traits and P-cycling dynamics. Mixed plantations significantly amplified labile P pools, particularly in the rhizosphere, through selective enrichment of *phoD*- and *pqqC*-harboring bacterial communities. Notably, plant properties positively regulated labile phosphorus in bulk soil, whereas soil properties predominantly influenced labile phosphorus in rhizosphere soil, indicating that both factors play crucial roles in mediating soil P transformation. These results advanced the understanding of plant-microbe-soil feedbacks in diverse forests, highlighting that tree diversity fosters functionally resilient microbial consortia capable of optimizing P utilization efficiency. By bridging microbial ecology with ecosystem nutrient cycling, this study provides support for adopting mixed-species afforestation as a sustainable strategy to mitigate P limitations in subtropical managed forests.

## SUPPLEMENTARY DATA

Supplementary data to this article can be found online.

## Data Availability

The raw sequencing data generated in this study have been deposited in the China National GeneBank DataBase (CNGBdb) under Accession No. CNP0008057. Soil phosphorus fractions data, ASV tables, and sequencing quality control data are available on FigShare: https://doi.org/10.6084/m9.figshare.29399921.
