# Supplementary material for: Coniferous-broadleaf mixed plantations reshape phosphorus-solubilizing bacterial communities and enhance soil phosphorus bioavailability in subtropical forests
Source: For Res (Fayettev). 2025 Oct 29;5:e022. doi: 10.48130/forres-0025-0023 (PMC12648022; doi:10.48130/forres-0025-0023)
Supplement: Supplementary file 1 — Supplementary data to this article can be found online. [file FR-2025-5-0023-Supplementary.zip › 10.48130_forres-0025-0023-Suppl-FigureS4.pdf]

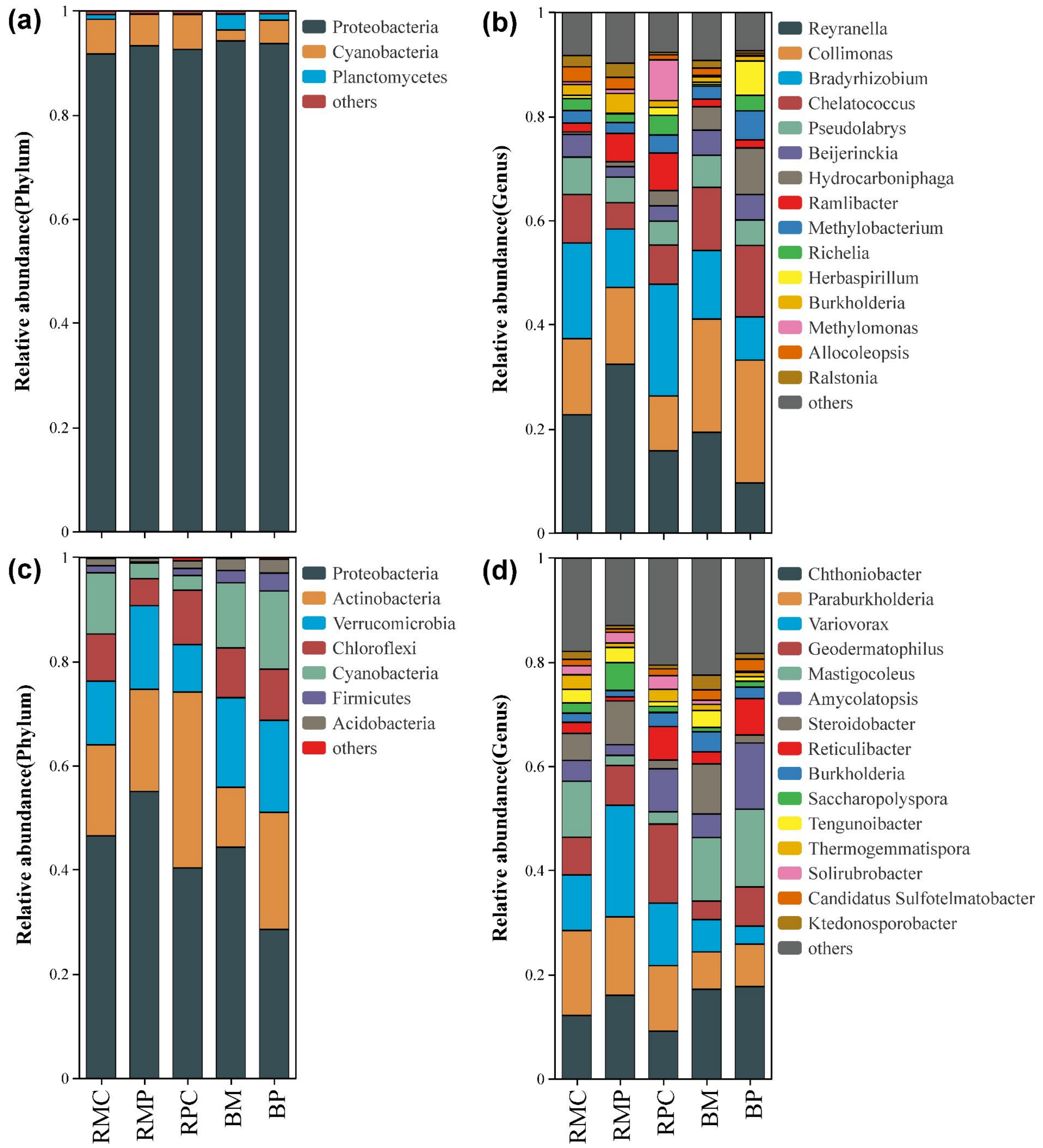

Figure S4 The *phoD*- and *pqqC*-harboring bacterial community composition in bulk and rhizosphere soil of different forest types. BM, bulk soil in mixed plantations; BP, bulk soil in monocultures; RMC, rhizosphere soil of *Cunninghamia lanceolata* in mixed plantations; RMP, rhizosphere soil of *Phoebe bournei* in mixed plantations; RPC, rhizosphere soil of *Cunninghamia lanceolata* in monocultures.
