# Supplementary material for: Coniferous-broadleaf mixed plantations reshape phosphorus-solubilizing bacterial communities and enhance soil phosphorus bioavailability in subtropical forests
Source: For Res (Fayettev). 2025 Oct 29;5:e022. doi: 10.48130/forres-0025-0023 (PMC12648022; doi:10.48130/forres-0025-0023)
Supplement: Supplementary file 1 — Supplementary data to this article can be found online. [file FR-2025-5-0023-Supplementary.zip › 10.48130_forres-0025-0023-Suppl-TableS1.pdf]

Table S1 Basic characteristics of experimental plots

Tree species	Tree height (m)	Diameter at breast height (cm)	Stand density (trees·ha^{-1})	Canopy density (%)	Dominant undergrowth vegetation species	Litter mass (g/m^2)
C.lanceolata	12.7	16.9	1800	78	*Maesa japonica, Tricalysia dubia, Woodwardia japonica, Dicranopteris dichotoma, Polypodiodes niponica*	102.3
C.lanceolata/ P. bournei	14.2<br>4.5	18.5<br>6.3	550<br>450	70	*Symplocos fukienensis, Ardisia punctata, Woodwardia japonica, Lindsaea orbiculate, Dicranopteris dichotoma, Millettia dielsiana*	156.2
