# Supplementary material for: Coniferous-broadleaf mixed plantations reshape phosphorus-solubilizing bacterial communities and enhance soil phosphorus bioavailability in subtropical forests
Source: For Res (Fayettev). 2025 Oct 29;5:e022. doi: 10.48130/forres-0025-0023 (PMC12648022; doi:10.48130/forres-0025-0023)
Supplement: Supplementary file 1 — Supplementary data to this article can be found online. [file FR-2025-5-0023-Supplementary.zip › 10.48130_forres-0025-0023-Suppl-TableS2.pdf]

Table S2 Effects of forest type and soil compartments and their interaction on the P fractions.

Parameters	*P*-value of linear mixed models			
	Forest type	Compartments	Type ×Compartments	Block
Labile P	0.019	**< 0.001**	**< 0.001**	0.996
Moderate labile P	**< 0.001**	0.195	**< 0.001**	0.988
Stable P	**< 0.001**	**< 0.001**	**< 0.001**	0.993
Total P	**< 0.001**	**0.001**	**< 0.001**	0.976
Organic P	**0.031**	**< 0.001**	**< 0.001**	1
Inorganic P	0.987	**< 0.001**	**< 0.001**	0.998

Note: The bold numerals indicate significance at $p < 0.05$ based on linear mixed models are presented.
