# Supplementary material for: Coniferous-broadleaf mixed plantations reshape phosphorus-solubilizing bacterial communities and enhance soil phosphorus bioavailability in subtropical forests
Source: For Res (Fayettev). 2025 Oct 29;5:e022. doi: 10.48130/forres-0025-0023 (PMC12648022; doi:10.48130/forres-0025-0023)
Supplement: Supplementary file 1 — Supplementary data to this article can be found online. [file FR-2025-5-0023-Supplementary.zip › 10.48130_forres-0025-0023-Suppl-TableS3.pdf]

Table S3 Effects of forest type and soil compartments and their interaction on the gene abundances and diversity of *phoD*- and *pqqC*-harboring bacterial communities.

Parameters	*P*-value of linear mixed models			
	Forest type	Compartments	Type ×Compartments	Block
Abundance of *phoD*	**< 0.001**	**0.001**	**< 0.001**	0.690
Abundance of *pqqC*	**0.005**	**< 0.001**	**< 0.001**	0.832
Chao of *phoD*	0.142	**< 0.001**	**< 0.001**	0.320
Shannon of *phoD*	0.415	**0.003**	**0.013**	0.558
Chao of *pqqC*	**0.050**	**< 0.001**	**< 0.001**	0.856
Shannon of *pqqC*	**0.006**	**< 0.001**	**< 0.001**	0.889

Note: The bold numerals indicate significance at $p < 0.05$ based on linear mixed models are presented.
