# Supplementary figures and images for: Coniferous-broadleaf mixed plantations reshape phosphorus-solubilizing bacterial communities and enhance soil phosphorus bioavailability in subtropical forests
Source: For Res (Fayettev). 2025 Oct 29;5:e022. doi: 10.48130/forres-0025-0023 (PMC12648022; doi:10.48130/forres-0025-0023)

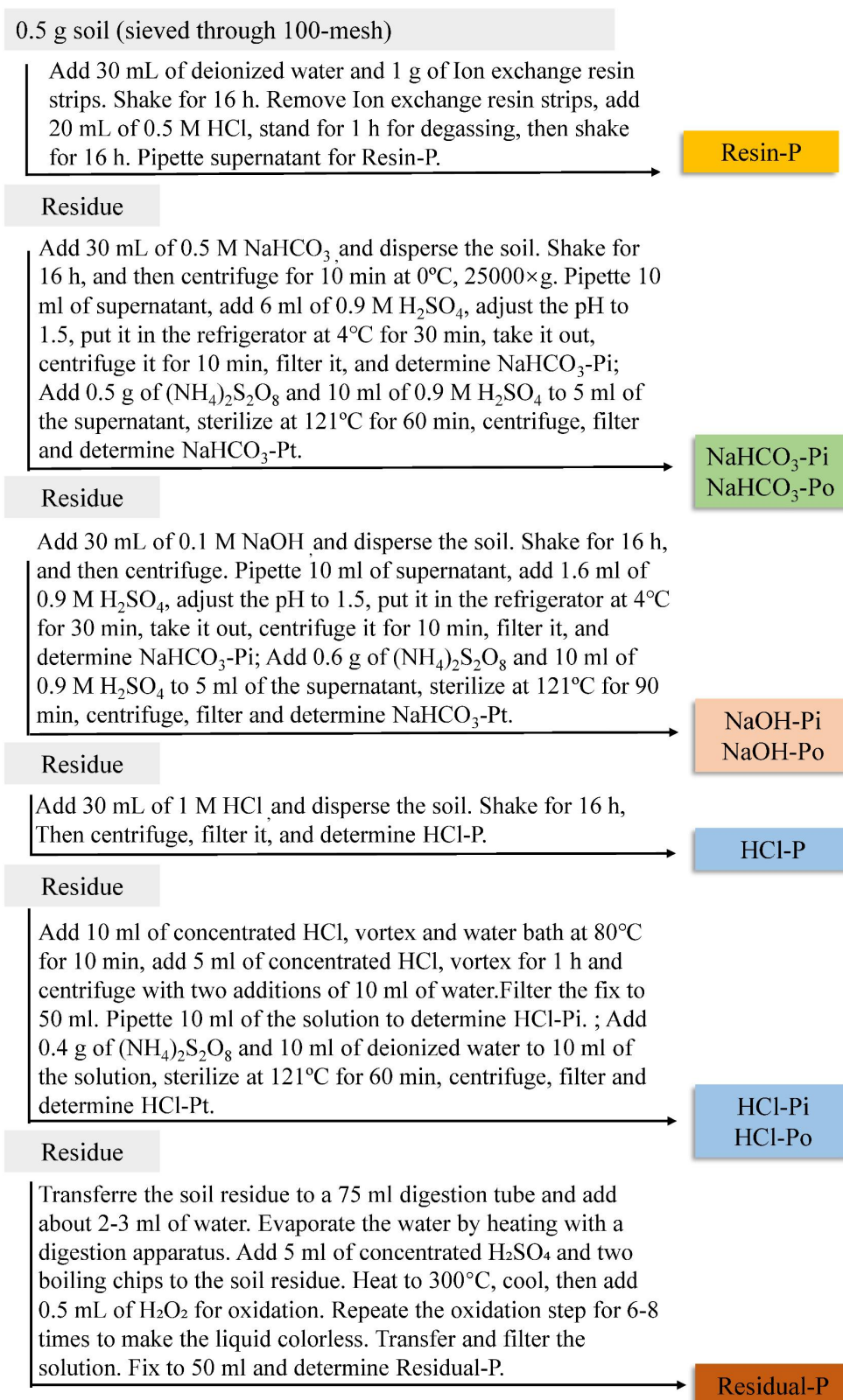

**Figure S1** Detailed extraction program for phosphorus fractions.

Supplement: Supplementary file 1 — Supplementary data to this article can be found online. [file FR-2025-5-0023-Supplementary.zip › 10.48130_forres-0025-0023-Suppl-FigureS1.pdf]
